# Psychosocial predictors of fear of cancer recurrence in a cohort of gynecologic cancer survivors

**DOI:** 10.1002/pon.6055

**Published:** 2022-10-28

**Authors:** Carlie A. Mell, Patricia I. Jewett, Deanna Teoh, Rachel I. Vogel, Susan A. Everson–Rose

**Affiliations:** 1 School of Nursing, University of Minnesota, Minneapolis, Minnesota, USA; 2 Department of Obstetrics, Gynecology, and Women’s Health, Division of Gynecologic Oncology, University of Minnesota, Minneapolis, Minnesota, USA; 3 Department of Medicine, Division of Hematology and Oncology, University of Minnesota, Minneapolis, Minnesota, USA; 4 Masonic Cancer Center, University of Minnesota, Minneapolis, Minnesota, USA; 5 Department of Medicine, Division of Geriatrics, Palliative, and Primary Care, and Program in Health Disparities Research, University of Minnesota, Minneapolis, Minnesota, USA

**Keywords:** anxiety, cancer, fear of cancer recurrence, gynecologic neoplasms, oncology, posttraumatic growth, psycho-oncology, psychosocial oncology, survivors

## Abstract

**Objective:**

To describe fear of cancer recurrence in a cohort of women with gynecologic cancers and to identify psychosocial predictors of elevated fear of recurrence.

**Methods:**

Survey data from an ongoing cohort study of gynecologic cancer survivors were used (*n* = 154). Relationships between fear of cancer recurrence measured by the 6-item Cancer Worry Scale in the most recent survey and psychosocial factors (cancer-related distress, depression, anxiety, hopelessness, and posttraumatic growth) assessed 6–18 months prior were examined using univariate and multivariate linear regression models, adjusting for age, cancer stage, cancer type, and time since diagnosis.

**Results:**

Most participants were ≥60 years old, diagnosed with early-stage cancer, and 2–5 years post-diagnosis. The mean score on the Cancer Worry Scale was 10.31 (SD = 3.01), and 46 individuals (30.0%) scored ≥12, indicating high fear of recurrence. In univariate analyses, greater distress (*p* = 0.007), anxiety (*p* = 0.006), hopelessness (*p* = 0.007), and posttraumatic growth (*p* = 0.0006) were significantly associated with higher scores on the Cancer Worry Scale. The associations of hopelessness and posttraumatic growth with higher Cancer Worry Scale scores remained significant after adjustment for covariates.

**Conclusions:**

Fear of recurrence is frequent among gynecologic cancer survivors. Women who reported more distress, hopelessness, anxiety and, surprisingly, more post-traumatic growth reported more fear. These results contribute to our understanding of which cancer survivors are most at risk of elevated fear of recurrence and highlight the importance of continued focus on psychosocial well-being among cancer survivors.

## 1 | BACKGROUND

As of 2019, there were approximately 16.9 million cancer survivors in the United States, among whom 1.3 million were gynecologic cancer survivors.^1^ The number of cancer survivors is increasing as the population ages and as detection and treatment of cancer improves.^1^ After primary treatment ends, cancer survivors continue to face a range of physical, psychological, social, and financial difficulties.^1^ In particular, gynecologic cancer survivors are understudied despite the potential for high morbidity. These survivors may have unique experiences, in part, due to the stigma associated with these cancers, especially for those with cervical, vaginal and vulvar cancers. Considering the growth of this population and its unique concerns, the needs and well-being of gynecologic cancer survivors deserve investigation. Notably, self-stigma, changes in self-concept, and psychological stressors have been linked with increased fear of cancer recurrence.^2,3^

Fear of recurrence is widely prevalent (39%–97%) and a great concern among cancer survivors.^4^ Fear of cancer recurrence can be distressing and long-lasting, and it is associated with reduced use of health services, lower adherence to follow-up recommendations, and lower quality of life.^5^ Therefore, identifying and supporting survivors at increased risk of elevated fear of cancer recurrence is pertinent. Fear of cancer recurrence can be defined as the tendency “to experience fear and worry about cancer returning after completing active treatment”.^6^ Several measures for assessing fear of recurrence exist, but the Cancer Worry Scale (CWS) is the most commonly used,^7^ and it has been translated and validated in multiple languages.^6,8^

Previous research has examined demographic and medical factors associated with fear of cancer recurrence. Younger age has been consistently linked to greater fear of recurrence, but research on other demographic factors has been mixed.^4^ Medical factors such as the type of cancer and the cancer stage at diagnosis influence risk of recurrence, but cancer stage and type may not equally drive fear of cancer recurrence.^9–12^

Psychosocial factors may also play a role in the development of fear of cancer recurrence. A recent model by Fardell et al. (2016) describes the multidimensionality of fear of recurrence, highlighting vulnerability factors that may contribute to heightened fear of cancer recurrence, such as the life impact of cancer; existential challenges; and emotional, behavioral, and cognitive responses to cancer.^2^ In line with what the model suggests, distress, depression, and anxiety have been linked to elevated fear of cancer recurrence in previous research, but much of this work has been cross-sectional and focused on breast cancer survivors.^13^ A related but distinct emotional factor is hopelessness, which has been independently linked to negative mental health outcomes,^14^ but with limited research regarding its relationship with fear of recurrence.^15^ Posttraumatic growth is a positive response to the existential challenges presented by traumatic experiences, such as cancer diagnosis and treatment. While evidence on posttraumatic growth and fear of cancer recurrence is also limited, the role of existential challenges in Fardell et al.’s model suggests that posttraumatic growth may protect against elevated fear of recurrence.

The two aims of this study were to describe the prevalence of fear of cancer recurrence in a cohort of gynecologic cancer survivors and to identify relationships between psychosocial variables and subsequent fear of cancer recurrence (6–18 months later). We hypothesized that greater distress, depression, anxiety, and hopelessness would be associated with greater fear, while greater posttraumatic growth would be associated with less fear of recurrence.

## 2 | METHODS

### 2.1 | Study design and population

The Gynecologic Oncology—Life After Diagnosis (GOLD) study is a prospective cohort survey study of patients seen within the University of Minnesota MHealth Fairview system. Participants were recruited March 2017 through March 2020. Eligible participants were English-speaking University of Minnesota patients who were 18 years and older with a history of a diagnosis of ovarian, cervical, uterine, vaginal, or vulvar cancer. Written informed consent was obtained from each participant prior to enrollment, and all participants signed a Health Insurance Portability and Accountability Act form allowing access to their medical record data for clinical data abstraction. The University of Minnesota Institutional Review Board (IRB#1612S01581) approved the GOLD study.

### 2.2 | Recruitment and data collection

Potential participants were identified using diagnosis codes using the electronic medical records. Eligible individuals were invited to participate during a clinic visit (March 2017–March 2020) or via mail (June–October 2019). Of the 814 individuals initially approached, 457 (56.1%) enrolled in the GOLD study. At time of the follow-up survey with data for this analysis (July 2020), 338 participants were alive and active participants; 48 deaths had occurred. A total of 249 (73.7%) women completed the July 2020 survey. For this analysis, we excluded women with missing data on the CWS (*n* = 21) as well as those for whom fear of recurrence may not be applicable, including those with any prior recurrence (*n* = 66) or unknown recurrence status (*n* = 3), and those with metastatic cancer (*n* = 5), resulting in a final analytic sample of *N* = 154.

Upon entering the study, participants completed a comprehensive baseline survey including demographics, self-reported clinical data, and emotional health measures. All surveys were administered on paper or online via REDCap,^16^ depending on participant preference. Clinical data—including comorbidities, cancer type, International Federation of Gynecology and Obstetrics stage at diagnosis, histology, and treatments received (chemotherapy, surgery, or radiation) and outcomes—were abstracted from electronic medical records. Follow-up surveys were administered approximately every 6 months and included emotional health measures and rotating topics of interest to the research team.

### 2.3 | Measures

#### 2.3.1 | Primary outcome

The primary outcome in this study was self-reported fear of cancer recurrence, which was assessed with a 6-item version of the CWS in the summer 2020 follow-up survey. The 6-item CWS has previously been reported to have acceptable reliability and validity^17^; in our study the Cronbach’s *α* = 0.86. Participants responded to questions about worries of recurrence and the impact of those worries on a 4-point Likert scale (1 = “never" to 4 = “almost always") with a possible score range of 6–24. Previous research suggests that a cut off of 12 (<11 vs. ≥12) is the most appropriate for identifying high fear of recurrence.^17,18^

#### 2.3.2 | Psychosocial predictors

Exposures of interest in this study were self-reported distress, depression, anxiety, hopelessness, and posttraumatic growth at a survey time point 6–18 months prior to the assessment of fear of cancer recurrence. If a participant completed multiple surveys within that time window, the survey used was the one closest to 1 year prior.

Cancer-related distress was measured using the 1-item National Comprehensive Cancer Network Distress Thermometer (DT) (score range 0–10; established cut-off score of 4 or greater suggests clinically relevant distress).^19,20^ Depression was measured using the Patient Health Questionnaire-8 (PHQ-8) (score range 0–24; Cronbach’s *α* = 0.87; established cut-off score of 10 or greater suggests clinically relevant depression symptoms).^21^ Anxiety was measured using the General Anxiety Disorder-7 (GAD-7) (score range 0–21; Cronbach’s *α* = 0.89; established cut-off score of 10 or greater suggests clinically relevant anxiety symptoms).^22^ A validated 2-item measure was used to assess hopelessness.^14^ Participants were asked to rate on a 5-point scale the extent to which they agree with the following statements: “The future seems to me to be hopeless, and I canť believe things are changing for the better” and “I feel it is impossible for me to reach the goals that I would like to strive for.” The potential score range is 0–8, with higher scores suggesting a greater degree of hopelessness, and there is no established cut-off; Cronbach’s *α* = 0.75. The Posttraumatic Growth Inventory-Short Form was used to measure posttraumatic growth.^23^ Participants rate the degree to which they experienced certain changes as a result of their cancer diagnosis, from 0 (“I did not experience this change”) to 5 (“I experienced this change to a very great degree”). Higher scores suggest greater subjective growth (range, 0–50) and there are no established cutoffs; Cronbach’s *α* = 0.96.

#### 2.3.3 | Demographic and socioeconomic variables

Covariates included in the multivariate analysis were identified a priori based on previous research. The chosen covariates (measured at baseline unless indicated otherwise) were: age at time of the summer 2020 survey (years), cancer site (endometrial, ovarian, cervical, or vaginal/vulvar), cancer stage (I/II or III), and time since gynecologic cancer diagnosis at time of the summer 2020 survey (years). Five individuals with stage IV disease were excluded from the analyses.

### 2.4 | Statistical analysis

Descriptive statistics were used to characterize the clinical, demographic, and psychosocial traits of the sample. Correlations among the psychosocial predictors were assessed. Univariate and multivariate associations were examined using linear regression analyses with continuous CWS scores as the outcome and continuous psychosocial scores as the main predictor variables. Multivariate models were adjusted for all covariates described above. Additionally, interactions between the psychosocial predictors and age were tested. These analyses were completed using the SAS version 9.4 procedures PROC FREQ, PROC MEANS, PROC CATMOD, PROC GENMOD, and PROC CORR. *p*-values less than 0.05 were deemed statistically significant.

## 3 | RESULTS

Table 1 shows participants' demographic and clinical characteristics of the 154 study participants included in the analysis. The mean age was 62.4 ± 10.4 years, most were non-Hispanic White (99.4%), 43.9% had at least a college degree, and 31.3% had a household income <$50,000. The most common primary cancer type was endometrial cancer (54.6%), followed by ovarian cancer (27.9%), cervical cancer (12.3%), and vaginal or vulvar cancer (5.2%). Most (76.8%) had early-stage cancer (FIGO stage I or II), and the majority (75.7%) were 2–5 years post-diagnosis.

The mean CWS score was 10.31 ± 3.01, with 46 (30.0%) of the participants scoring ≥12, indicating high fear of recurrence. Means and standard deviations of the psychosocial measures are shown in Table 2. Twenty seven of the participants (20.2%) reported clinically meaningful distress (≥4 on DT), 9 (6.5%) reported clinically relevant depression symptoms (≥10 on PHQ-8), and 11 (7.9%) reported clinically relevant anxiety symptoms (≥10 on GAD-7).

The correlations between the psychosocial predictors are presented in Table 3. Distress, depression, anxiety, and hopelessness were significantly associated with each other; posttraumatic growth was not correlated with any of the other psychosocial predictors.

Unadjusted linear regression analyses showed that greater distress (*p* = 0.007), anxiety (*p* = 0.006), hopelessness (*p* = 0.007), and posttraumatic growth (*p* = 0.0006) scores were associated with greater subsequent CWS scores (Table 4). Participants with greater depressive symptoms tended to have greater CWS scores, but the association was not significant (*p* = 0.08). After adjusting for covariates, greater hopelessness (*p* = 0.04) and posttraumatic growth (*p* = 0.005) remained significantly associated with greater CWS scores. Each 1-point increase in hopelessness was associated with a 0.24-point greater CWS score, and each 1-point increase in posttraumatic growth was associated with a 0.05-point greater CWS score. This equates to 0.50-point and 0.70-point increases in CWS per 1-standard deviation increase in hopelessness and posttraumatic growth, respectively. The regression coefficient for anxiety showed that a 1-point increase in anxiety was associated with a 0.13-point greater CWS score (*p* = 0.06). A sensitivity analysis that additionally adjusted for the time between the survey when the exposures were measured and the 2020 cross-sectional survey showed no meaningful changes in the estimated associations (data not shown). Both age (univariate association, *p* = 0.004; adjusted models, *p* ≤ 0.01 in all models) and years since diagnosis (univariate association, *p* = 0.03; *p* > 0.05 in all adjusted models) were inversely associated with fear of recurrence. No evidence was found for an interaction between age and any of the psychosocial predictors.

## 4 | DISCUSSION

Fear of cancer recurrence is a common concern among cancer survivors. This study utilized survey data from two time points to examine fear of recurrence and psychosocial predictors among gynecologic cancer survivors who had not experienced a recurrence. We hypothesized that greater distress, depression, anxiety, and hopelessness would predict greater fear of recurrence and that greater posttraumatic growth would predict lower fear of recurrence. Our results partially supported our hypotheses.

The psychosocial profile of this cohort was positive overall, consistent with previous research indicating a generally high quality of life of gynecologic cancer survivors long-term.^24^ The distress, depression, and anxiety scores for most participants fell below established thresholds for clinical relevance. The brief hopelessness scale and the Posttraumatic Growth Inventory both lack established cutoff scores. However, 77.9% of participants reported a hopelessness score between 0 and 2, which would indicate little or no hopelessness. The Posttraumatic Growth Inventory has previously been used to study posttraumatic growth in adult survivors of breast cancer and head and neck cancer.^25–27^ The mean score in our sample was comparable but lower than in these prior studies, so it is possible that our sample experienced less posttraumatic growth; however, based on current evidence and without established score cutoffs, it is difficult to conclude if this difference is meaningful. As for fear of recurrence, approximately one third (30%) of participants reported high fear of cancer recurrence, which is consistent with the findings of a previous review of fear of recurrence in adult cancer survivors.^4^ The proportion of participants reported as having elevated fear of recurrence varies, but in other studies as well as in this cohort, it is a common concern.^4^

The relationships between fear of cancer recurrence and the selected psychosocial predictors were largely as expected based on the literature and our conceptual model, with some exceptions. Depression is often found to be associated with fear of cancer recurrence.^4,28^ In our study, women with greater depressive symptoms tended to report greater fear of recurrence, but this association was not statistically significant in univariate or multivariate analyses. While reasons for this are unclear, our sample, on average, was more than 2 years post-diagnosis and so while fears of recurrence may still have been present, depressive symptoms were not prominent. Additionally, prior studies differed from ours in terms of type of cancer studied and length of time between cancer diagnosis and assessment of depression and fear of recurrence. Other research suggests that persons with clinically meaningful fear of recurrence do not commonly meet criteria for psychological disorders.^29^ Thus, it may be that depression and fear of recurrence are unique constructs that only sometimes overlap in cancer patients' experience. Distress and anxiety have also been identified as strong correlates of fear of recurrence.^4^ In our study, distress and anxiety were associated with fear of recurrence in univariate analysis, but not in the multivariate analysis, suggesting that the associations with fear of recurrence largely were explained by traditional risk factors, and especially age, which was a statistically significant covariate in the adjusted models.

The most unexpected result was the observed relationship between greater posttraumatic growth and increased fear of cancer recurrence. Posttraumatic growth is considered a positive response to the existential challenge posed by cancer, and we expected it to be inversely associated with fear of recurrence. Few studies examining posttraumatic growth in cancer survivors have been published, and fewer still have reported on both posttraumatic growth and fear of cancer recurrence. Even so, this positive association is consistent with findings from some recent studies^30,31^; however, the reasons for this observation are not immediately obvious. The relationship between posttraumatic growth and existential fears may be more complex than we hypothesized. One recent study examined the components of posttraumatic growth and found that greater growth in new possibilities, greater spiritual change, and greater appreciation of life were all associated with greater fear of recurrence.^31^ Iťs possible that post-traumatic growth may heighten one’s sense of inevitable eventual losses, which may then increase fear of cancer recurrence. In other studies, posttraumatic growth was inversely associated with depressive and anxiety symptoms in cancer survivors.^32^ In our study, though, posttraumatic growth was not significantly correlated with distress, depression, anxiety, or hopelessness. The clinical relevance of this observed association between posttraumatic growth and fear of recurrence is unclear since the estimated effect size was small. Nevertheless, because posttraumatic growth is understood as a positive outcome that supportive professionals may seek to foster in cancer survivors, these observed relationships deserve further investigation.

It is important to note that several protective psychosocial characteristics - which were not addressed in this study - may be influencing results. Recent studies of women with breast cancer have highlighted the importance that hope, optimism, self-efficacy, and social problem-solving skills have in enhancing resilience, promoting positive coping, and contributing to greater post-traumatic growth.^33,34^ It is quite plausible that these psychosocial resources would help women who have experienced gynecologic cancers also cope with the fears they experience about recurrence. Future studies should expand the scope of psychosocial assessments to include these types of resources, particularly to explore the unexpected observation that greater post-traumatic growth was related to more fear of recurrence.

While not our main topic, our study also confirmed previous findings that younger age was associated with greater fear of recurrence. Additionally, our results indicate a weak or nonexistent relationship between clinical and disease characteristics and fear of recurrence. Cancer site and stage were neither significantly associated with fear of cancer recurrence in linear regression analyses nor significant covariates in multivariate models. While perhaps surprising, this finding is consistent with previous research of gynecologic cancer survivors^10^ and general cancer survivors.^9^

### 4.1 | Study limitations

One limitation is that it is unknown whether the CWS accurately captures clinically significant fear of cancer recurrence, which limits the strength of conclusions we can draw. Consistent and accurate assessment of clinically meaningful fear of cancer recurrence is an ongoing challenge in this area of research.

Further limitations arise from the study structure, the composition of the cohort, and the methods. The data for this study came from cross-sectional surveys, and fear of cancer recurrence was only assessed at one time point, limiting our ability to draw conclusions about trajectory and causal relationships. Further, this study is based on self-reported data, which raises questions of potential self-report bias. This limitation is tempered by the fact that we used well-validated self-report measures that are commonly used in large cohort studies; moreover, patient reports of their own psychosocial functioning are widely regarded as accurate. Nonetheless, using additional observation methods (e.g., clinician-report or peer ratings) on some measures may provide added value and mitigate any self-report bias that may be present. The study population included only survivors of gynecologic cancers, most of them older, non-Hispanic White women; thus, our results may not generalize to other diagnostic or demographic groups. Most participants were ≥2 years post-diagnosis, so our findings may not apply to those more recently diagnosed with gynecologic cancer. Generalizability may be further restricted by the initial recruitment response rate and subsequent participant loss to follow up due to death and non-participation in the follow-up survey. We compared baseline characteristics of the study population with eligible non-responders to the follow-up survey and with all non-responders (Supporting Information Table AS1). Completers were largely similar to eligible non-responders, with the exception that completers were less likely to have ovarian cancer (*p* = 0.03).

### 4.2 | Clinical implications

Screening for distress, including fear of recurrence, and referral for individualized psychosocial support is the recommended approach for distress management by the National Comprehensive Cancer Network.^20^ The cancer care team can manage mild distress by explaining the risks of recurrence and normalizing feelings of fear of recurrence. This study highlights the importance of discussing fears with patients regardless of prognosis. We found that greater distress, anxiety, hopelessness, and posttraumatic growth—and also younger age—may be indicators that could help identify patients most likely to experience elevated fear of recurrence. In combination with screening tools and conversations with clinicians, this knowledge can aid in connecting survivors with interventions that have been shown to reduce fear of cancer recurrence, such as group and individual cognitive behavioral therapy, gratitude interventions, and counseling from specialist nurses.^5^ Further research is needed, however, to optimize the screening and referral approach and identify evidenced-based interventions most acceptable to this patient population.

## 5 | CONCLUSION

In this study of gynecologic cancer survivors, nearly one third of participants reported high fear of recurrence. Using survey data from two time points, our data suggest that greater distress, hopelessness, anxiety, and posttraumatic growth may be associated with greater fear of recurrence approximately 1 year later. The relationship between posttraumatic growth and fear of recurrence was unexpected and merits further investigation. Future studies with a larger sample exploring the possibly causal relationship among fear of recurrence and post-traumatic symptoms and post-traumatic growth may be warranted. These findings contribute to our understanding of the predictors of fear of recurrence and emphasize the role of psychosocial well-being in the care of cancer survivors.

## Supplementary Material

Table S1

## Figures and Tables

**TABLE 1:** Demographic and clinical characteristics

Characteristic	*N*	%
Age group, years^a^		
<40	6	3.9
40–49	14	9.1
50–59	27	17.5
60–69	77	50.0
≥70	30	19.5
At least college degree		
No	83	56.1
Yes	65	43.9
Missing	6	
Annual household income		
<$50,000	46	31.3
$50,000–99,999	55	37.4
≥$100,000	35	23.8
Prefer not to say	11	7.5
Missing	7	
Partner status		
Single/divorced/widowed	52	35.4
Married/partnered	95	64.6
Missing	7	
Race/ethnicity		
Non-Hispanic White	153	99.4
Other	1	0.7
Employment status		
Full or part time	80	54.1
Not working	15	10.1
Retired	53	35.8
Missing	6	
Cancer site		
Ovarian	43	27.9
Cervical	19	12.3
Endometrial	84	54.6
Vaginal/vulvar	8	5.2
Stage		
I or II	116	76.8
III	35	23.2
Missing	3	
Time since diagnosis^a^		
<2 years	13	8.6
2–5 years	115	75.7
≥5 years	24	15.8
Missing	2	

a Characteristics as reported at study entry, except age and time since diagnosis (July 2020 survey).

**TABLE 2:** Psychosocial measures

Measure	*N*	Mean (SD)	Possible range^a^
1. Cancer Worry Scale	154	10.31 (3.01)	6–24
2. Distress Thermometer	134	2.16 (2.50)	0–10
3. Depression (PHQ-8)	139	3.32 (4.01)	0–24
4. Anxiety (GAD-7)	139	2.88 (3.56)	0–21
5. Hopelessness	140	1.24 (2.08)	0–8
6. Posttraumatic Growth Inventory	136	21.71 (13.95)	0–50

a Threshold for clinical relevance: CWS ≥12, Distress Thermometer ≥4, PHQ-8 ≥10, GAD-7 ≥10. No established cutoff for Hopelessness or Posttraumatic Growth Inventory.

**TABLE 3:** Correlations among psychosocial predictors

	Distress	Depression	Anxiety	Posttraumatic growth
Depression	0.57***			
Anxiety	0.58***	0.66***		
Posttraumatic growth	0.06	−0.06	−0.01	
Hopelessness	0.20*	0.36***	0.25**	−0.04

* <0.05

** <0.01

*** <0.0001.

**TABLE 4:** Results of univariate and multivariate analyses

Parameter^a^	Univariate				Multivariate^b^			
	Coefficient	95% CI		*p*-value	Coefficient	95% CI		*p*-value
Distress	0.28	0.08	0.48	**0.007**	0.18	−0.02	0.37	0.08
Depression	0.11	−0.01	0.23	0.08	0.03	−0.09	0.16	0.30
Anxiety	0.19	0.06	0.33	**0.006**	0.13	0.00	0.26	0.06
Posttraumatic growth	0.06	0.03	0.09	**0.0006**	0.05	0.01	0.08	**0.005**
Hopelessness	0.32	0.09	0.56	**0.007**	0.24	0.02	0.47	**0.04**

*Note:* Bolded values are statistically significant in our analyses.

a Per additional one unit increase in score.

b Adjusted for age, cancer site, disease stage, and time since diagnosis.

## Data Availability

The data that support the findings of this study are available on request from the corresponding author. The data are not publicly available due to privacy or ethical restrictions.
