# Supplementary material for: Psychosocial predictors of fear of cancer recurrence in a cohort of gynecologic cancer survivors
Source: Psychooncology. 2022 Oct 28;31(12):2141–8. doi: 10.1002/pon.6055 (PMC9798429; doi:10.1002/pon.6055)
Supplement: Supplementary file 1 — Table S1 [file PON-31-2141-s001.docx]

*Supplementary Table A: Comparison of study population, eligible non-responders, and all excluded participants*

|  | **Study population (N=154)** | **Non-responders excluding those ineligible (those with a recurrence or metastatic cancer) (N=96)** | | **All excluded (including those with a recurrence or metastatic cancer) (N=184)** | |
| --- | --- | --- | --- | --- | --- |
| **Characteristics at baseline** | **N (%)** | **N (%)** | **P-value*** | **N (%)** | **P-value*** |
| *Age group, years* |  |  | 0.26 |  | 0.58 |
| <40 | 6 (4) | 8 (9.3) |  | 14 (8.2) |  |
| 40-49 | 18 (11.8) | 7 (8.1) |  | 16 (9.4) |  |
| 50-59 | 39 (25.7) | 26 (30.2) |  | 42 (24.6) |  |
| 60-69 | 66 (43.4) | 37 (43) |  | 74 (43.3) |  |
| >=70 | 23 (15.1) | 8 (9.3) |  | 25 (14.6) |  |
| Missing | 2 | 10 |  | 13 |  |
| *At least college degree* |  |  | 0.98 |  | 0.52 |
| No | 83 (56.1) | 47 (56) |  | 99 (59.6) |  |
| Yes | 65 (43.9) | 37 (44.1) |  | 67 (40.4) |  |
| Missing | 6 | 12 |  | 18 |  |
| *Annual household income* |  |  | 0.23 |  | 0.18 |
| 1 | 46 (31.3) | 31 (36.5) |  | 62 (37.6) |  |
| 2 | 55 (37.4) | 27 (31.8) |  | 50 (30.3) |  |
| 3 | 35 (23.8) | 15 (17.7) |  | 32 (19.4) |  |
| Prefer no to say | 11 (7.5) | 12 (14.1) |  | 21 (12.7) |  |
| Missing | 7 | 11 |  | 19 |  |
| *Partner status* |  |  | 0.08 |  | 0.11 |
| Not partnered | 52 (35.4) | 39 (47) |  | 72 (44.2) |  |
| Partnered | 95 (64.6) | 44 (53) |  | 91 (55.8) |  |
| Missing |  | 12 |  | 21 |  |
| *Race/Ethnicity* |  |  | 0.07 |  | 0.08 |
| Other | 1 (0.7) | 4 (4.2) |  | 7 (3.8) |  |
| NH White | 153 (99.4) | 92 (95.8) |  | 176 (96.2) |  |
| Missing | 0 | 0 |  | 1 |  |
| *Employment status* |  |  | 0.37 |  | 0.61 |
| Full or part time | 80 (54.1) | 51 (60) |  | 86 (51.5) |  |
| Not working | 15 (10.1) | 11 (12.9) |  | 23 (13.8) |  |
| Retired | 53 (35.8) | 23 (27.1) |  | 58 (34.7) |  |
| Missing | 6 | 11 |  | 17 |  |
| *Cancer site* |  |  | **0.03** |  | **0.0002** |
| Ovarian | 43 (27.9) | 36 (37.5) |  | 86 (46.7) |  |
| Cervical | 19 (12.3) | 19 (19.8) |  | 27 (14.7) |  |
| Endometrial | 84 (54.6) | 34 (35.4) |  | 58 (31.5) |  |
| Vaginal/Vulvar | 8 (5.2) | 7 (7.3) |  | 13 (7.1) |  |
| *Stage* |  |  | 0.09 |  | **<0.0001** |
| I | 101 (66.9) | 50 (53.2) |  | 67 (37.0) |  |
| II | 15 (9.9) | 11 (11.7) |  | 22 (12.2) |  |
| III | 35 (23.2) | 33 (35.1) |  | 67 (37.0) |  |
| IV | 0 | 0 |  | 25 (13.8) |  |
| Missing | 3 | 2 |  | 4 |  |
| *Time since diagnosis* |  |  | 0.81 |  | 0.29 |
| <1 year | 51 (34) | 26 (30.2) |  | 49 (28.7) |  |
| 2 | 37 (24.7) | 26 (30.2) |  | 50 (29.2) |  |
| 3 | 59 (39.3) | 32 (37.2) |  | 63 (36.8) |  |
| 4 | 3 (2) | 2 (2.3) |  | 9 (5.3) |  |
| Missing | 4 | 10 |  | 14 |  |

*compared with study population
